# Exploring of new natural Saudi nanoparticles: investigation and characterization

**DOI:** 10.1038/s41598-020-78429-5

**Published:** 2020-12-09

**Authors:** Refat El-Sheikhy

**Affiliations:** Bghshan Research Chair in Expansive Soil, Civil Engineering Department, Faculty of Engineering, King Saud University, Riyadh, Saudi Arabia

**Keywords:** Solid Earth sciences, Engineering, Materials science, Nanoscience and technology

## Abstract

A new natural nanomaterial in Saudi Arabian soil has been explored. It is green nanoclay consisting of two-dimensional nanoparticles with special properties and dimensions. These nanoparticles are called Saudi halloysite-like nanotubes (SHNTs) because they are similar to halloysite nanotubes (HNTs). SHNTs are transparent having a special cross-section in polygonal shapes, such as hexagonal, with unique dimensions in comparison to that of HNTs. Additionally, external width of SHNTs is 20–50 nm, length is 50–600 nm and can reach 10,000 nm while lumen width of SHNTs is larger than that of HNT, along with SHNTs having thinner walls; these attributes make SHNTs good as nanocontainer. Surface area of SHNTs (168 m^2^/g) is larger than surface area of HNTs (65 m^2^/g). SHNTs are bendable with a slight curvature, while HNTs are always straight. Geometry, dimensions, microstructure, chemical composition, surface area and zeta potential of SHNTs are characterized using SEM, TEM, EDX, Langmuir surface area technique, a laser particle size analyser and ZP analysis. SHNTs have many applications in industrial, medical and advanced nanocomposite production. Experimental work has been carried out on nanocomposites made of HDPE reinforced with 5% SHNTs, proving enhancements in mechanical, fracture and thermal properties of original HDPE materials.

## Introduction

**Figure 1:**
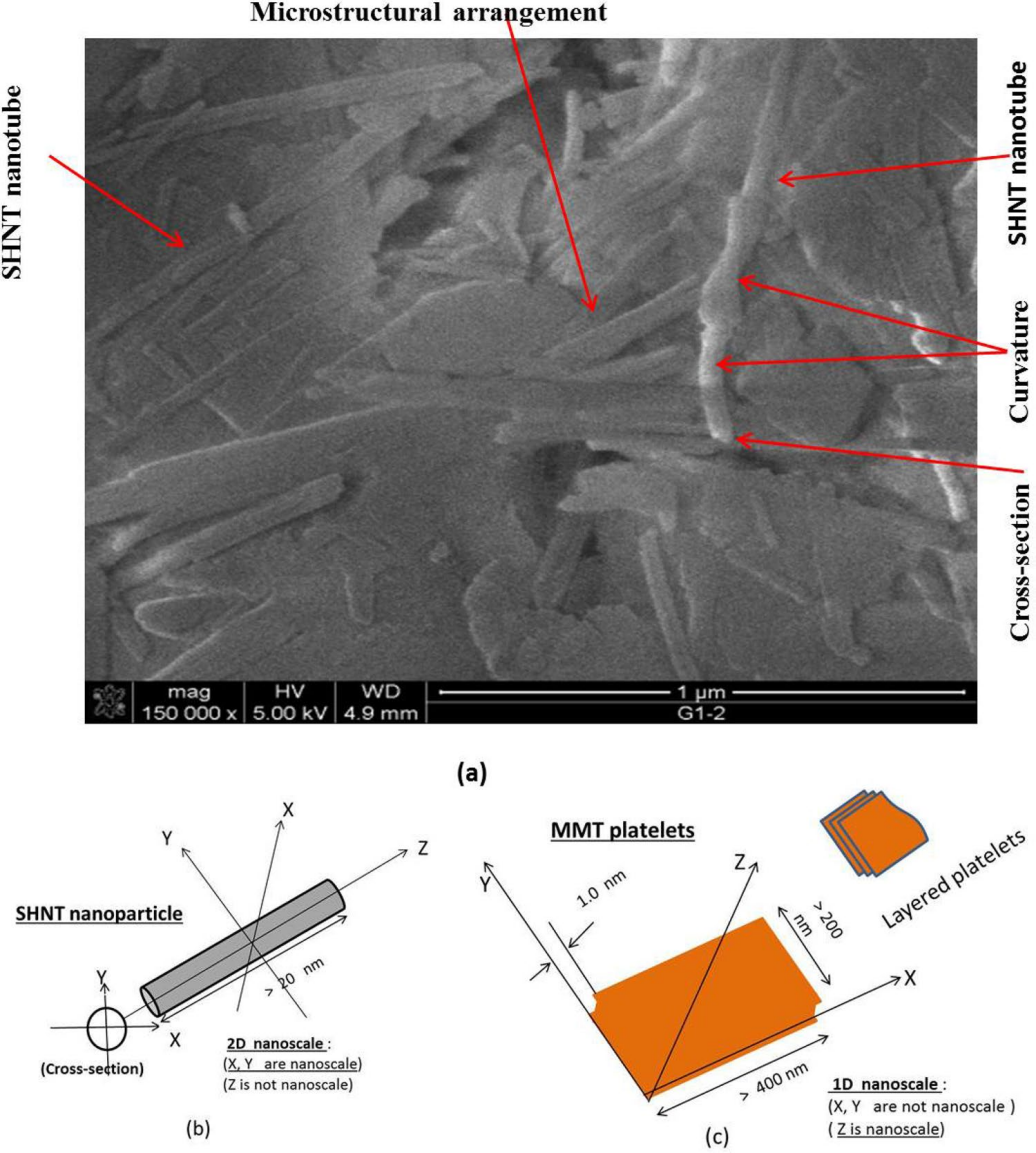
SEM image showing the SHNT microstructure, dimensions, geometry, cross-section and curvature.

**Figure 2:**
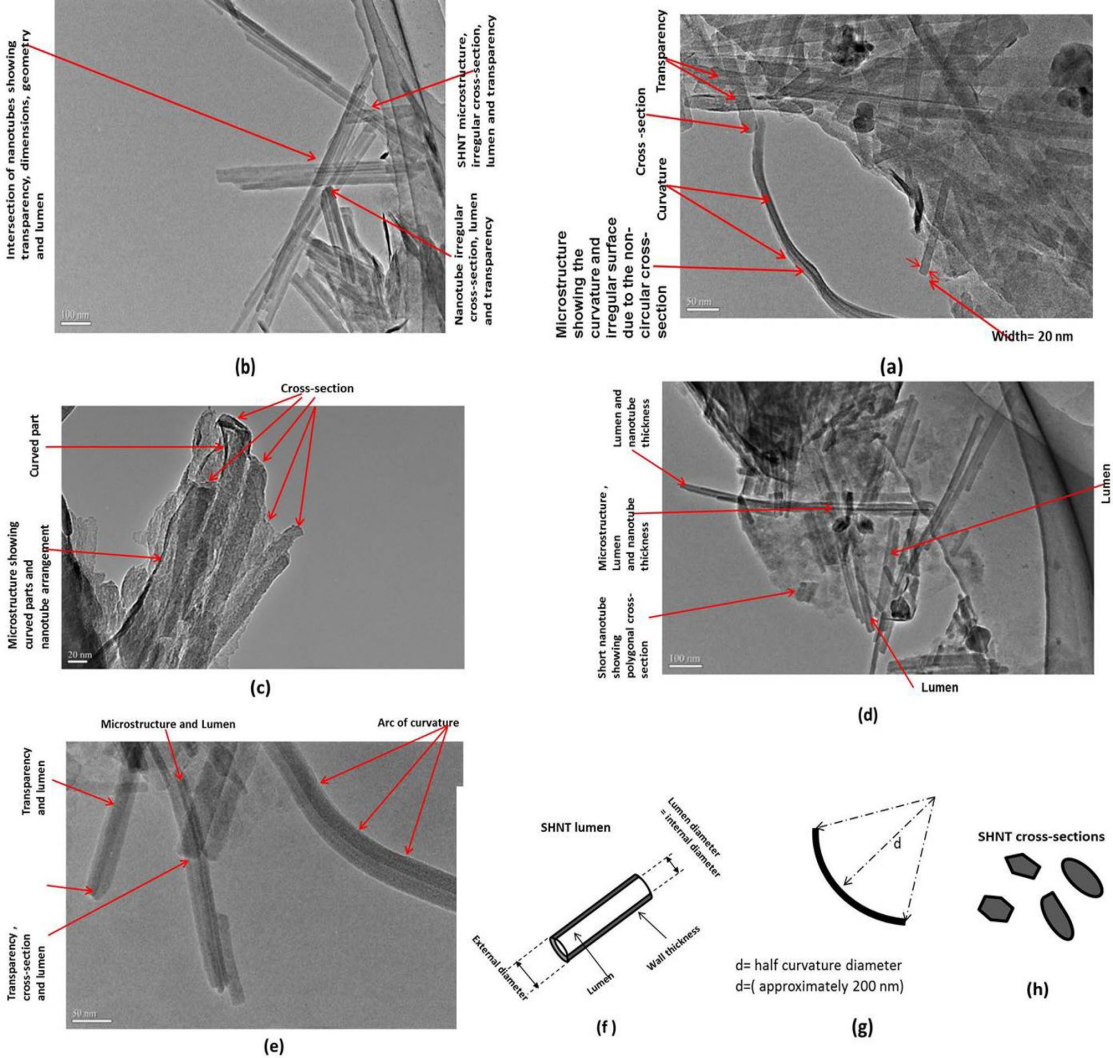
TEM micrograph showing different aspects of the SHNTs.

**Figure 3:**
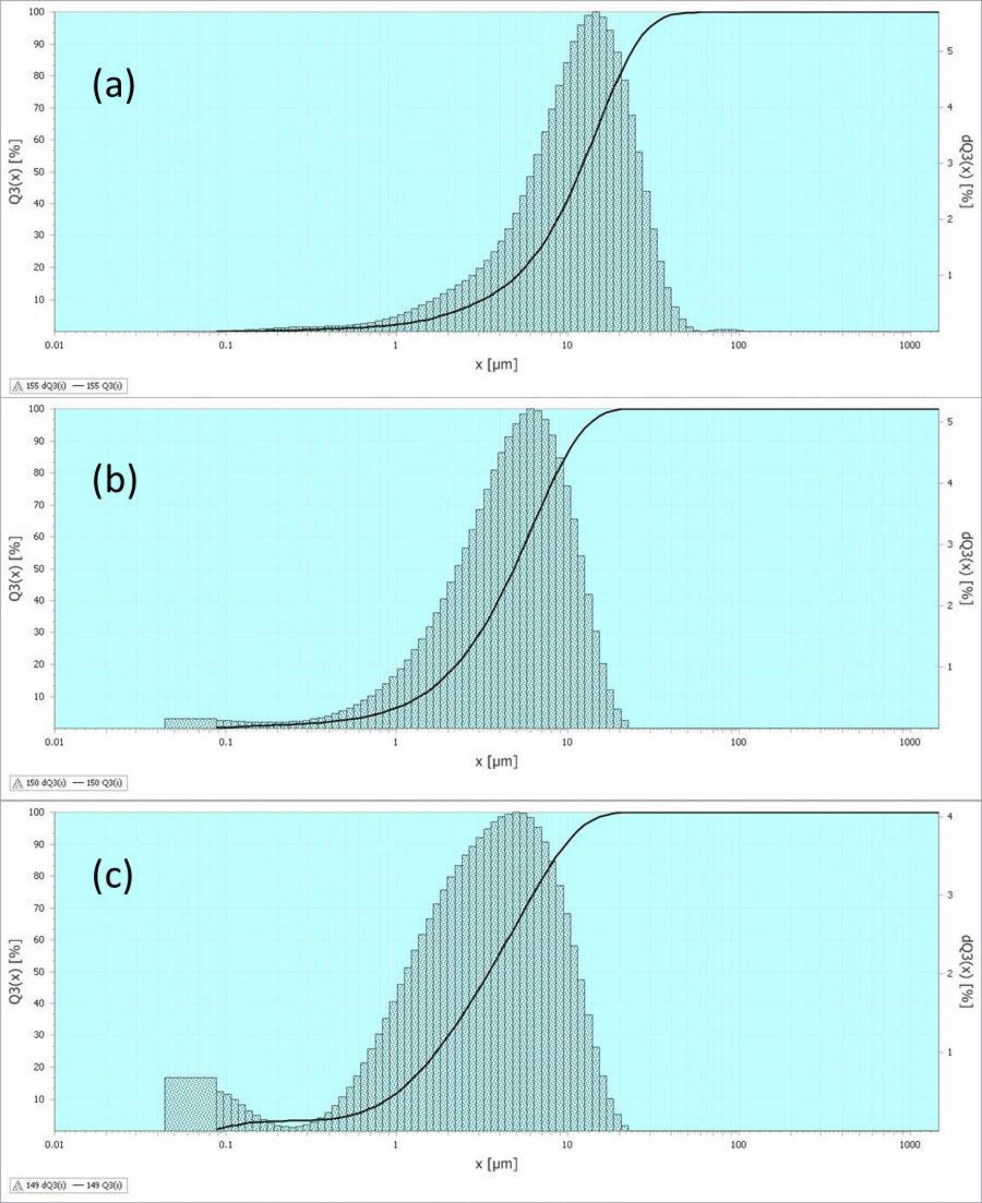
Results of the mean particle size analysis by the laser particle analyser for (**a**): MMT, (**b**): kaolinite, (**c**): SHNT (device measuring range: 100–20,000 nm).

It is known that clay minerals have several conventional types^1^. There are some different types that can be considered rare earth materials^1–10^. Conventional clays, such as smectite and kaolinite families, have almost same structure and main chemical compositions, where main chemical structure consists of silicate aluminium. The properties of conventional clay types can be easily characterized, including their crystal data, physical properties, geometries, dimensions, chemical compositions, surface areas, and microstructural morphologies. Conventional clay types are hydrophilic and have ability to absorb water and water vapor. Some conventional clay types have high volumes and shape-changing abilities, demonstrating high swelling or shrinking ratios, such as MMT clay, which is called expansive clay^1^. Particles of expansive clay types have strong bonds between clay layers, producing an agglomeration of large particles. The structure of conventional clay types consists of agglomerated platelet-shaped layers. On the other hand, there are rare types known as tubular nanoclays, such as halloysite HNTs and imogolite, which have different structures, shapes and properties^2–10^. These tubular clay types do not expand and do not have platelet or layered shapes. Halloysite is a rare clay found in certain places around the world. Generally, conventional clay minerals, such as montmorillonite MMT, consist mainly of Al and Si layers. Halloysite consists of two layers in the shape of nanotubes with an external diameter of 50 nm and a lumen with a diameter of 15 nm and a length of 1–1250 nm, where the external layer is Si and the internal layer is Al with the presence of H_2_O between the two layers^2,3^. The HNT surface area is 65 m^2^/g, and specific weight is 2.53 g/cm^3^. The chemical composition is (Al_2_Si_2_O_5_(OH)_4_·2H_2_O). In our current research, we explored another rare and naturally green tubular nanoclay type with new properties similar to those of halloysite, which we called Saudi halloysite-like nanotubes (SHNTs). These SHNTs have special dimensions and geometries with good purity. SHNTs can be used as nanocontainers, nanoreactors and in many industrial, environmental and medical applications Additionally, SHNTs can be classified as two-dimensional (2D) nanoparticles (X and Y directions are nanoscale but Z direction may be nanoscale or microscale). In comparison to conventional nanoclay, MMT particle is a one-dimensional (1D) nanoscale particle (only the Z direction is nanoscale, while the X and Y directions are microscale) since MMT thickness is 1.0 nm, while MMT width is 100–200 nm, and the length is 400 nm, whereas 3D nanoparticles indicate that particles are spherical with nanoscale sizes in all directions (the X, Y and Z directions are nanoscale). Therefore, MMT is 1D nanoscale, while SHNTs are 2D nanoscale, as shown in Fig. 1. Some types of one-dimensional nanoclays like MMTare always expansive, swelling easily due to absorbing water or shrinking easily due to drying. HNT particles are opaque, while SHNTs are transparent. Generally, conventional nanoclay is used as a filler material to enhance properties of other materials, such as polymers, thereby producing new nanocomposites called CPNCs^11–13^. The SHNTs microtructure and dimensions are different from the HNT microstructure. In addition, geometry, surface area, cross-section, diameter, lumen and shape are different. SHNT is not expansive since its structure, surface area and shape cannot permit the absorption or retention of water between particle interfaces. The SHNT surface area is large (approximately 168 m^2^/g). Furthermore, SHNTs are transparent exfoliated particles without any agglomeration, as shown in Figs. 1 and 2. Additionally, the dimensions of SHNTs are unique, as shown in Figs. 1, 2 and 3. These dimensions can enhance the properties of the SHNT based polymer nanocomposite better than that of other nanoclays, such as MMT platelets.

## Results

### Scanning electron microscopy (SEM) analysis

Detailed characterization using scanning electron microscopy (SEM) was carried out to study SHNTs. SEM micrograph provides characterization details of the microstructure of this newly explored nanoclay in Saudi Arabia called SHNTs. Figure 1 shows the composition, shape, structure, and dimensions of SHNTs, where external diameter is approximately 20–50 nm and length starts from 20 to approximately 600 nm. SHNT particles are shown in clear separate cases as straight nanotubes with a slight curvature, which means that they are not bonded to each other. SHNT particle aspects, such as shape, geometry, dimensions, appearance, arrangement, structure and morphology can be seen in Fig. 1a. The particles are clearly shown as nanotubes without having a definite arrangement or oriented direction. SHNT length and dimensions can be easily measured from SEM images. In comparison to other types of nanoclay, such as MMT, it is known that MMT always agglomerates and forms large particles, while SHNT clay particles can easily be seen as separate particles, as shown in Fig. 1a. There is no bonding between the SHNT particles, while MMT particles usually bond to each other, producing agglomerated microparticles, as shown in Fig. 1c. Nanotube clay particles are classified as two-dimensional (2D) nanoparticles, where X and Y directions are nanoscale, while Z direction may be nanoscale or microscale, as shown in Fig. 1b. On the other hand, nanoclay platelets, such as MMT, are classified as one-dimensional (1D) nanoparticles since X and Y directions are large, while Z direction is 1.0 nm, as shown in Fig. 1c.

### Transmission electron microscopy TEM analysis

TEM characterization can investigate the most important aspects of microstructural morphology in addition to physical properties and elemental composition of these newly explored SHNTs. TEM images in Fig. 2a–e include all the discovered data on SHNTs where each image explains some important aspects and properties with clear dimensions. Additionally, the data are not only qualitative but mostly quantitative due to the detailed and accurate measurements, which match SEM data and results of other characterization techniques (EDX, ZP and particle size analysis). The images show the shape and geometry; arrangement; intersection; transparency; quasi-circular and quasi-polygonal cross-section; dimensions, including length, lumen diameter, external diameter, and wall thickness; and curvature. The cross-section is not 100% circular or rounded, but contains rectangular, polygonal and elliptical shapes, as shown in Fig. 2. These polygonal cross-sections can be curved as shown in Fig. 2. The diameter of the nanotube is between 15 and 50 nm, while length is approximately 50–600 nm.

### SHNTs microstructure

Regarding investigation of SHNT shape, geometry, curvature, dimensions, cross-section and surface, microstructure is investigated by both SEM, as shown in Fig. 1, and TEM, as shown in Fig. 2. The geometries of cross-section and curvature are clear, where cross-section seems polygonal and nanotubes are clearly shown with arcs and curvature. Usually, cylindrical tubes are difficult to bend or curve, while rectangular or polygonal sections can be bent or curved, as shown in the current case of SHNTs (Figs. 1, 2). The shape is a nanotube with a width of 20–50 nm, a length of 50–10,000 nm, and a surface area of 168.7 m^2^/g. Each image in Fig. 2 indicates some important aspects and measurements, where Fig. 2a shows that microstructural shape of the particle is a longitudinal tubular nanoparticle, and this nanotube is transparent. Additionally, irregular polygonal cross-section is shown in Fig. 2h. Moreover, straight and curved SHNT particles are shown with a curvature of radius 2d = 400 nm in Fig. 2g. The irregular external surface is due to irregular polygonal cross-section in which the cross-section is not 100% circular. The fine polygonal dimensions include a particle length starting from 50 to approximately 600 nm and a width or external diameter of approximately 20–50 nm, while analysis by laser analyser shows that particle length may be as long as 10,000 nm. Figure 2b explains each of the microstructural shapes of the particle. Figure 2c shows each microstructural shape of the particle, including polygonal cross-section, curved parts of SHNT particles and the particles in a non-regular arrangement. Figure 2c also shows the non-bonded SHNT particles explaining that microstructure consists of single SHNT particles, which also proves that SHNTs do not swell because it is not a highly hydrophilic material. This behaviour can be explained by particles always appearing as separated single particles without any bonds between them, thus exhibiting a high degree of exfoliation and dispersion. Therefore, SHNTs do not have a certain particle arrangement. Figure 2d,e show that lumen has a tubular shape in straight and curved particles. The lumen diameter starts from 15 nm up to the SHNT particle wall thickness, which equals the difference between the SHNT external diameter and the internal diameter or lumen diameter (Wall thickness = External diameter − Internal diameter). For example, the SHNT wall thickness = 20 nm − 15 nm = 5 nm, which represents a single-walled SHNT particle. In another example, SHNT wall thickness = 50 nm − 15 nm = 35 nm, which actually represents thickness of multi-walled SHNT particles. In addition, Fig. 2f–h explains important aspects of the SHNT particles. Figure 2f shows the 2D nanoscale SHNT particle, including its particle orientation, cross-section, lumen, internal diameter, external diameter and wall thickness, while Fig. 2g shows SHNT particle curvature in addition to the length of the radius of curvature, which is approximately 400 nm. In addition, Fig. 2h shows potential cross-sectional shapes of SHNTs.

### Energy dispersive analysis of X-rays (EDX) of SHNTs

The investigation and exploration of elemental and chemical composition of these newly discovered Saudi natural nanoparticles is essential for current study to understand the material type, properties and potentially suitable applications. Since both SEM and TEM have ability to investigate chemical composition of the materials using the electron beam and well-known crystal properties of elements listed in the data library of the microscope memory. The results of the chemical composition using SEM and TEM are almost identical, but TEM–EDX is better by showing detailed elements that SEM–EDX cannot observe. SEM–EDX shows that chemical composition of SHNTs contain 47.56% oxygen O, 8.1% aluminium Al, 30.48% silicon Si and 13.64% calcium Ca, while chemical analysis by TEM–EDX shows that SHNTs contain 48.31% oxygen O, 10.15% aluminium Al, 30.57% silicon Si and 1% calcium Ca in addition to 2.66% potassium K, 3.76% magnesium Mg and 3.57% ferrite Fe. TEM–EDX is more believable since the TEM chemical composition is purer than that of SEM–EDX sample, although the results are almost identical. EDX proves that the cross-section is almost circular since Al ratio is half of Si ratio when Al is the internal layer, while Si is the outer layer where Al/Si = (1/2). Since it is natural, it includes some other elements with an 87% purity, which is a good ratio, especially since no purification process was conducted; additionally no additives were supplied to prevent changes during analysis. Other tested samples have purity ratios near 100%. It is shown from the analysis that (Al:Si) ratio is 8.31:30.48, which can be simplified to 1:3.67, thereby matching SHNT structure since it consists of two layers where the inner layer is (Al) (smaller ratio) while the outer layer is (Si) (larger ratio). This result means that SHNT structure matches the chemical composition. On the other hand, SEM–EDX predicts the chemical composition shown in Table 1 with a purity ratio of approximately 89.07% without the purification process, indicating that Al:Si ratio is approximately 1:3.01, while TEM–EDX ratio is 1:3.67. The two ratios are almost the same.

**Table 1:** Energy dispersive analysis of X-rays (EDX) of the newly explored SHNTs: (1) TEM–EDX, (2) SEM–EDX.

(1) TEM–EDX		(2) SEM–EDX	
Element	wt%	Element	wt%
Oxygen O	48.31	Oxygen O	47.56
Aluminium Al	10.15	Aluminium Al	8.1
Silicon Si	30.57	Silicon Si	30,48
Calcium Ca	1.0	Calcium Ca	13.64
Potassium K	2.6		
Magnesium Mg	3.76		
Ferrite Fe	3.57		
Total wt%	100%	Total wt	100%

### Particle size analysis of SHNTs

It is important to check the particle size of the newly explored SHNTs to investigate the maximum and minimum dimensions of the particles in addition to their uniformity and distribution in comparison to other similar materials such as conventional halloysite nanotubes HNTs and conventional montmorillonite MMT. SHNTs, MMT and Saudi kaolinite clay particle sizes are tested using the available device, which is a wet analysis laser particle size analyser. The nanoclay analysis using a wet system is better than that using a dry system since the wet analysis depends on providing a very small sample of the material in a large amount of distilled water with continuous ultrasonic mixing to disperse and exfoliate the bonded particles. Then, the size of the particles is measured by using infrared and laser beams. The infrared and laser beams measure global particle size, uniformity and percentage ratio in the sample. It should be noted that the available device can measure only in the range of 100–20,000 nm. Therefore, this instrument can provide data about maximum size of SHNT particles, while minimum size can be measured through SEM and TEM analyses. The results are shown and compared in Fig. 3, indicating that maximum size of the SHNTs is approximately 10,000 nm, while minimum size is 15 nm, as measured by TEM. Figure 3 shows the SHNT particle distribution, gradient and size percentage in comparison to HNTs and MMT. It is clear from Fig. 3 that SHNTs have the best gradient, distribution and dimensions. The smallest microstructural dimensions are measured by SEM, and TEM as shown in Figs. 1 and 2. These results prove and confirm surface area result of SHNTs, where it is larger than surface area of conventional HNTs.

### Surface area of SHNTs

The surface area of nanoparticles is the most important property for applications, especially for nanocomposites where interfacial bond between nanoparticles and other materials depends on contact surface area. Therefore, surface area of SHNTs is tested using standard Langmuir method for SHNTs and agglomerated MMT. The tested surface area of SHNTs is 168.23 m^2^/g larger than that of HNTs with an increase of 258%, in which the known surface area of HNTs is 65 m^2^/g^3^. The results are compared to the known surface area of exfoliated MMT (800 m^2^/g)^1^ and conventional MMT (91 m^2^/g). It is difficult to exfoliate MMT since it always agglomerates at the microscale due to its high hydrophilic ability of absorbing atmospheric water, while SHNTs do not agglomerate. The high surface area of SHNT particles can be attributed to some important reasons related to the dimensions, shape, cross-section and appearance, where the high length (20–10,000 nm) and irregular shape of the cross-section cause the external surface of the SHNT particles to increase the surface area. In addition, the individual appearance of SHNT particles as single particles without agglomeration can certainly increase the surface area. At the same time, a high surface area can confirm the microstructural aspects regarding cross-section, distribution, arrangement, shape, exfoliation, dimensions and geometry. Therefore, the surface area and microstructure prove each other.

### Zeta potential (ZP) for SHNTs

**Figure 4:**
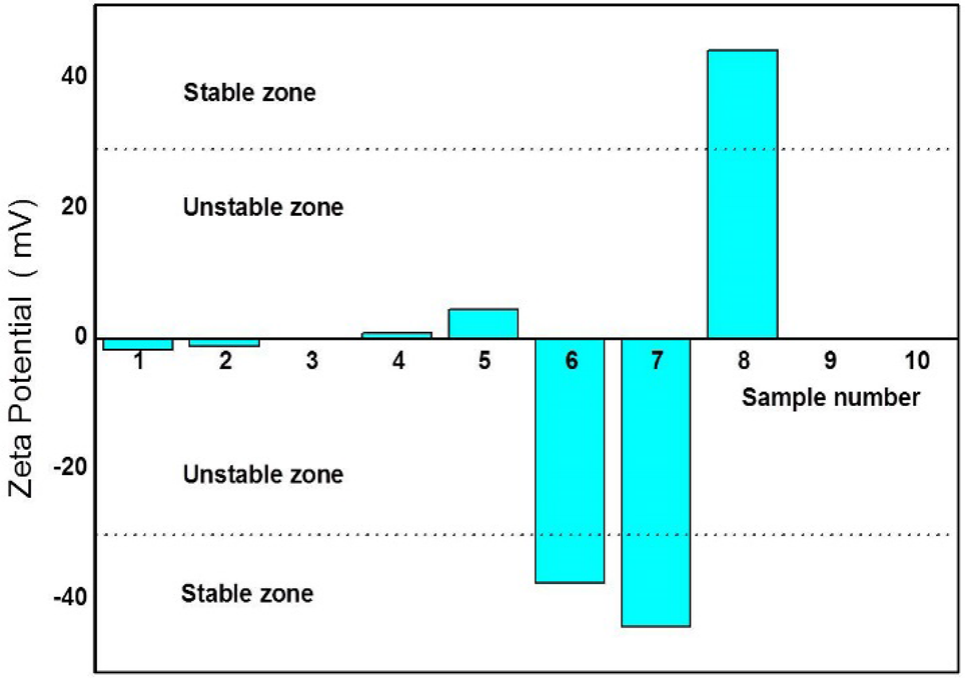
Results of the zeta potential test.

ZP test results are shown in Fig. 4, along with the electrochemical changes and stabilities of SHNTs. Zeta potential tests were carried out for different samples, including natural Saudi kaolinite samples and samples of MMT nanoclay (samples 1–5), in addition to samples of the current materials of natural Saudi clay nanotubes (SHNTs) (samples 6, 7). Each test was carried out three times. The average ZP result was considered for each sample. A high ZP proves that the particles exhibit electrical stability without agglomeration, while a low ZP means particle agglomeration will occur. It is shown in Fig. 4 that ZP for SHNTs is between a moderate stability of ZP = − 37.5 mV and a good stability of ZP = − 44.2 mV, which means that SHNTs have good electronic stability in water and that there will be no agglomeration. ZP value of other kaolinite is between 0.1 and − 15 mV, clearly indicating that conventional kaolinite undergoes rapid coagulation aggregation (ZP = 1.7 mV), rapid flocculation aggregation (ZP = 0.1–1.2 mV) and emerging instability (ZP = − 15.1 mV). The comparison proves that SHNTs have good electronic stability in water and that there is no agglomeration due to the presence of high repulsion between the SHNT particles, which means that SHNT clay is not expansive clay. Therefore, in water, it will not easily sediment, but will take a long time to sediment based on its own weight. Therefore, SHNTs sample will still be able to be suspended, while other samples will quickly sediment. All ZP samples were prepared in distilled water with a pH of 7.0 for testing under the same conditions: at room temperature (25 °C) and tested at the same time by the same person. The ZP test also proved that kaolinite and MMT clay can agglomerate easily to form large particles, which can sediment rapidly in a short time based on its velocity due to its size and dimensions in addition to its absorption of water.

### Swelling–shrinking phenomenon

It is clear that SHNTs have no ability to swell; thus their ability to swell and shrink is non-existent based on ZP results. These results show good electrical stability of SHNTs in addition to their geometry, shape, dimensions, microstructure and chemical composition. Therefore, the newly explored natural Saudi halloysite-like nanotube (SHNT) particles are not expansive soil.

### Mechanical and fracture properties of SHNT-based CPNCs

**Figure 5:**
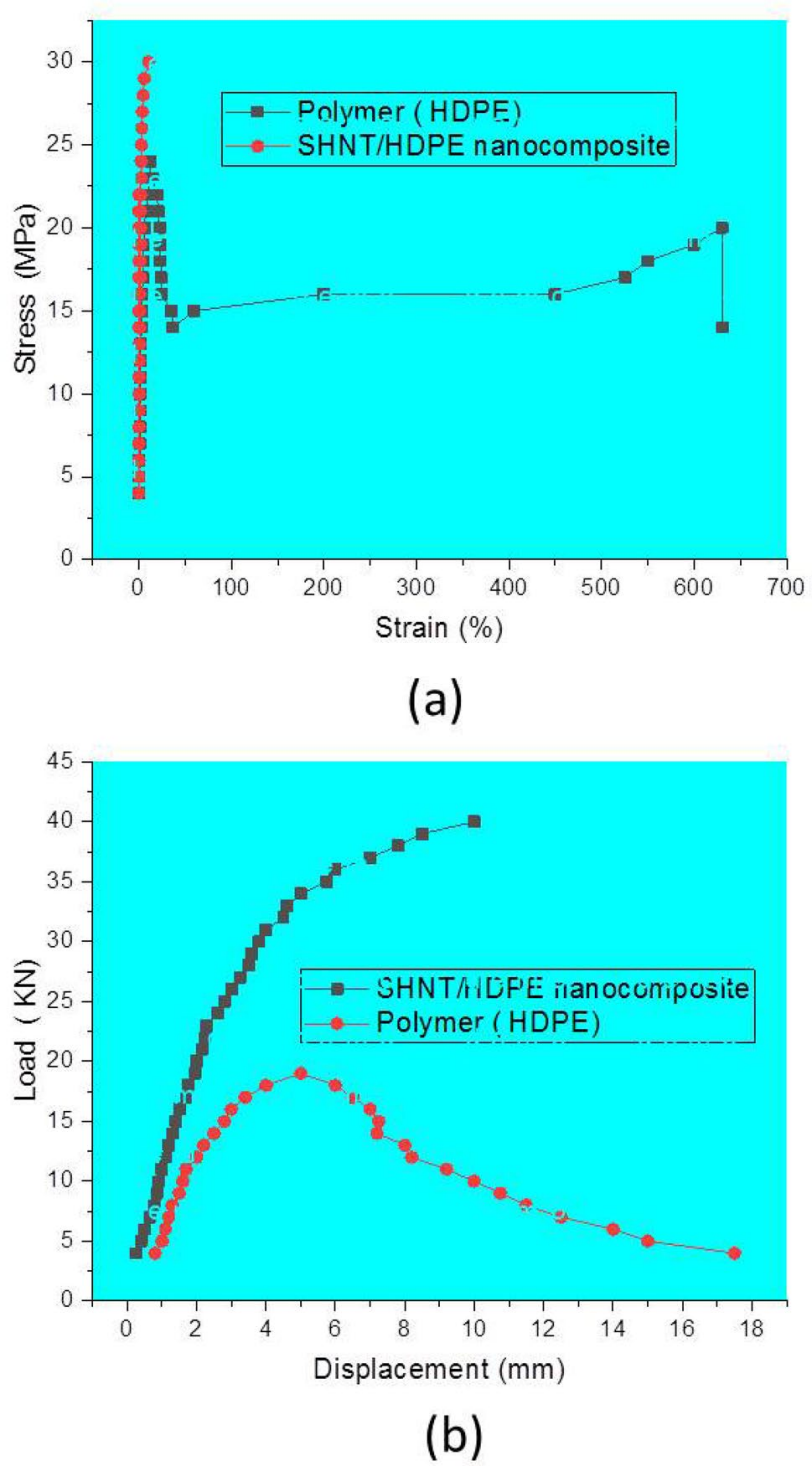
(**a**) Stress–elongation relationship of the tensile test for pure HDPE based on standard test ASTM D638, and the stress–elongation relationship for the tensile CPNC samples made of HDPE and the newly explored SHNT at a ratio of 95:5 by weight (the test was based on ASTM D638). (**b**) Stress–elongation relationship of the flexural test for pure HDPE based on the standard test ASTM D5045, and the stress–elongation relationship for the flexural test samples of the CPNC made of HDPE and the newly explored SHNT at a ratio of 95:5 by weight (the test was based on ASTM D5045).

The results of the tests are shown in Table 2 and Fig. 5a, including the mechanical test for tensile strength (σ test) and other mechanical properties, such as Young’s modulus of elasticity (E) and Poisson’s ratio (ν), for both pure HDPE and the CPNC made of HDPE and SHNT based on the ASTM standard D638^14^. Figure 5b and Table 2 show the results of the fracture mechanics test to measure the fracture toughness (K_Q_) or critical stress intensity factor (K_Ic_) of pure HDPE and CPNC made of HDPE and 5% SHNT dry powder mixed in an extruder under a high temperature of 250 °C. The results explain the effect of SHNTs on the enhancement in mechanical properties and fracture resistance. The reasons for this enhancement are due to SHNTs reinforcing HDPE due to a high bi-interfacial bond between the surface area of the SHNT nanotubes and the HDPE matrix. A high interfacial bond is generated due to the polygonal cross-section and the large surface area. The test samples were prepared based on the ASTM D638 test as a standard dumbbell shape for mechanical property testing to check the tensile strength, Young's modulus of elasticity, Poisson's ratio, and elongation. The tested samples for fracture resistance were prepared according to ASTM D5045^15^. Regarding the results of ASTM D638^14^ for the tensile strength test and ASTM D5045 for the three-point load test for fracture properties, it is found that adding 5% SHNTs to the HDPE matrix produces a CPNC that converts ductile HDPE to ductile–brittle nanocomposites or brittle nanocomposites, as clearly shown in Fig. 5a. From the curves relating stress and strain, HDPE shows a linear elastic stage followed by a large-scale yielding zone followed by a large-scale plasticity zone with large-scale deformation at a strain of 630%. In contrast, the test curve of the CPNC made of HDPE and 5% SHNT, shown in Fig. 5b, represents a brittle, pure linear elastic nanocomposite. Additional comparison results are provided in Table 2, where the elongation decreases by 30.9% at yield and 98.25% at failure with a decrease in Poisson's ratio (ν) by 55.5%. The mechanical, flexural and fracture properties change when tensile strength increases by 23.4% at yield and 83.2% at failure, Young's modulus of elasticity (E) increases by 49%, flexural strength increase by 87.4% at yield and by ten times at failure, while fracture toughness decreases by half. The fracture properties and fracture toughness test are carried out based on ASTM D5045, where the fracture toughness (KQ = K_Ic_) is estimated from the standard test ASTM D5045^15^ based on the following equation

$$ [{\text{K}}_{{{\text{Ic}}}} = {\text{K}}_{{\text{Q}}} = [{\text{P}}_{{\text{Q}}} /{\text{BW}}^{{0.{5}}} ]{\text{ F}}\left( {\text{x}} \right)]. $$

where F(x) = 6 x^½^ (1.99 − x (1 − x) (2.15–3.93x + 2.7 x^2^))/(1 + 2 x)(1 − x)^3/2^, x = (a/W), B = specimen thickness, P = critical load P_Q_, W = specimen height, and a = crack length.

**Table 2:** Mechanical and fracture properties of HDPE and the CPNC.

Mechanical test (tensile) (ASTM D638), and fracture test (flexural 3point load) (ASTM D 5045)	HDPE (pure)	CPNC (SHNT + HDPE)	Change percentage (% )
Tensile strength at yield (MPa), **ASTM D638**	23.9	**29.5**	+ 23.4
Tensile strength at break (failure) (MPa), **ASTM D638**	15.5	**28.4**	+ 83.2
Elongation at yield (elastic elongation) (%), **ASTM D638**	11	**8.4**	− 30.9
Ultimate elongation (elastic–plastic) (%), (at failure) **ASTM D638**	630	**11**	− 98.25
Young’s modulus (E) (MPa), **ASTM D638**	1188	**1771**	+ 60
Poisson's ratio (ν), **ASTM D638**	0.45	**0.2**	− 55.5
Max. flexural strength (MPa), **ASTM D5045** (3-point test load)	18.5	**34.67**	+ 87.4
Flexural modulus (MPa), **ASTM D5045**, (3point test load)	691.9	**1224**	+ 76.87
Flexural stress at break (fracture), (MPa) , **ASTM D5045**	3.72	**40.9**	+ 1000
Fracture toughness K_Ic_, MPa (mm)^0.5^ **ASTM D 5045**	123	**60**	− 51.2
Material TYPE	Ductile viscoplastic material	Ductile–brittle viscoelastic nanocomposite	Conversion from to brittle–ductile nanocomposite
**(+) = **Increase , (−) = Decrease **CPNC (SHNT + HDPE) = **(95% HDPE + 5% **SHNTs**)

### Thermal resistance of SHNTs

**Figure 6:**
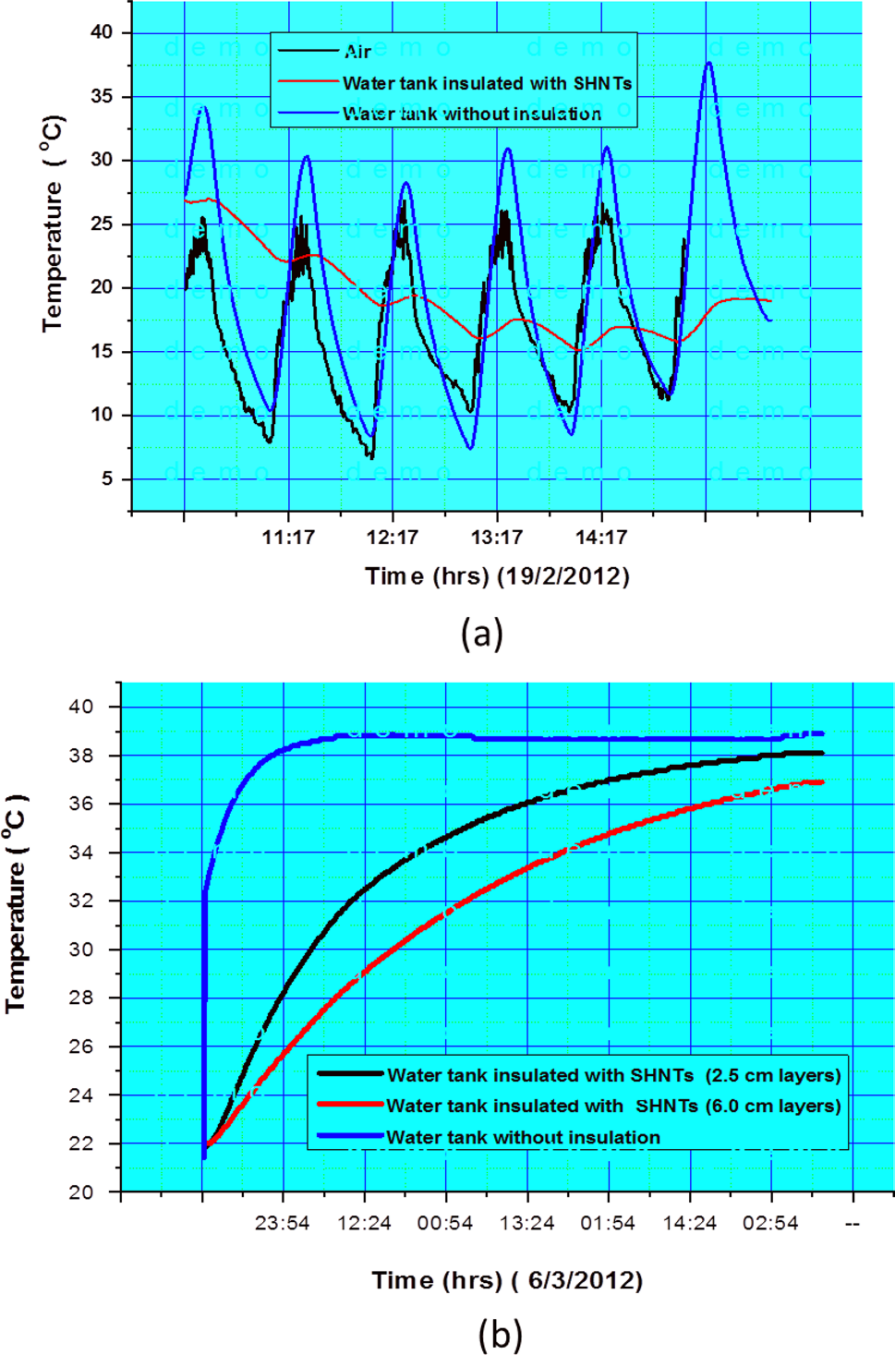
(**a**) Relationship between the time and temperature changes of water in the tank insulated with SHNTs in comparison to the open-air temperature and water in the tank without insulation under natural temperature changes in the field. (**b**) Relationship between the time and temperature changes of water in the tank insulated with SHNTs in comparison to water in the tank without insulation at a constant temperature of (40 °C) in the control room of the laboratory.

Since the thermal conductivity coefficient (k) of conventional clay is variable (0.15–1.8 W/m K) depending on the humidity, moisture, degree of saturation and surrounding atmospheric temperature, it will be very difficult to detect k exactly for the SHNT clay due to the presence of lumens. The Standard k for dry conventional clay is 0.15, but of course, hollow clay nanotubes will have a lower thermal conductivity factor than conventional clay. Therefore, we experimentally checked the effect of a real case of SHNTs as an insulator for the water temperature contained in a 0.2 mm thick high-density polyethylene HDPE container with a 20 L volume. The standard k of high density polyethylene is 0.5 W/m K. The results show that a thin layer of a dry SHNT powder can maintain the water temperature when the atmospheric temperature changes are between 3 and 37 °C, the water temperature change without isolation is between 3 and 37 °C, and the temperature of the insulated water is 15–18 °C. The test results under variable temperatures during the day and night are shown in Fig. 6a. The measurements are carried out in open air. Since the results are important for Saudi Arabia in regard to saving water and energy, after the success shown by these results, large water tank models were made of the CPNC consisting of HDPE reinforced by 5% SHNT powder to be used in Saudi houses. These water tanks allow water temperature to decrease from 37 °C in the day to 18 °C and keep it at 15 °C when atmospheric temperature decreases to 3 °C at night. Thus, water use is comfortable in all seasons, especially winter, and electrical energy is saved in regard to heating water in winter and reducing the water temperature in summer to be at the level of safe human use without injury or pain. Testing thermal resistance ability of SHNTs at a constant temperature (40 °C) was carried out in a control room. The results are shown in Fig. 6b. The results show that the water temperature of the non-insulated water tank reaches 37 °C after just 4 h, while the water temperature of the insulated water tank using SHNT reaches 37 °C after 3 days, which means that the tank insulated by SHNT has a thermal resistance for temperature changes with the following ratio 72/4 (18 times more insulated) when compared to the non-insulated tank.

## Discussion

This research explored, characterized and tested a new rare earth material in Saudi soil, which is a natural green nanotube clay with excellent properties called Saudi halloysite-like nanotubes (SHNTs). These SHNTs have special dimensions, geometries, physical properties, mechanical properties, thermal properties and fracture properties. SHNTs can be found in good purity, and they are suitable for producing special high-quality nanocomposites with a high grade of mechanical properties, fracture properties and thermal resistance for different medical, industrial and environmental applications. The cross-section of SHNTs is quasi-circular, as shown in Figs. 1 and 2, in which the cross-sections are not perfectly circular but closer to hexagonal, pentagonal or elliptical shapes. SHNTs are clearly transparent and the particles and objects near the bottom can be seen through the particles above them, especially at particle intersections. The dimensions are unique, where the external dimension equals 20–30 nm with a lumen width of 15–20 nm and a length of 50–600 nm; the maximum length is 10,000 nm. The internal diameter of the lumen seems larger than other types of known halloysite-like clay with thicknesses less than other types. Therefore, it is easy to recognize it as transparent. It can be filled with other materials, thereby functioning as nanocontainers. SHNT surface area is 168 m^2^/g, which is larger than surface area of HNTs (65 m^2^/g). SHNTs do not swell like MMT. SHNTs have a straight shape in addition to a slightly curved shape. SHNTs are a brittle and inorganic thermosetting material with a large surface area. The SHNT dimensions and microstructural analysis, particles size analysis, chemical analysis, surface area analysis and zeta potential analysis have been investigated and characterized using SEM, as shown in Fig. 1; TEM, as shown in Fig. 2; EDX, as shown in Table 1; the Langmuir surface area technique, laser particle size analyser, as shown in Fig. 3; and ZP analysis, as shown in Fig. 4. Notably, the results match each other. In comparison to other types of clay, SHNTs have unique properties, as shown in Table 2. In addition, effects have been investigated for each of the mechanical properties shown in Table 2 and Fig. 5a based on ASTM D638, while the flexural and fracture properties shown in Table 2 and Fig. 5b are based on ASTM D5045. Moreover, thermal property experiments were conducted and their results were investigated, as shown in Fig. 6. The comparison proves that SHNTs are similar to HNTs in some aspects, such as their clay family and chemical composition, but are different and better in other important aspects, such as their physical properties, namely, their microstructure, surface area, geometry and dimensions; furthermore, SHNTs are completely different from MMT in all aspects. SHNTs can enhance the properties of a polymer matrix better than other nanoclay platelets since SHNTs do not agglomerate like MMT nanoclay platelets. The comparison explains the advantages of SHNTs, in which they have a large surface area, long length, polygonal cross-section, curvature, ability to bend without breaking, large lumen and transparency. A large surface area with a polygonal cross-section increases the bi-interfacial bond between SHNTs and other matrices for nanocomposite production, thereby increasing the enhancement of the mechanical and fracture properties. A long length in the presence of a high surface area increases the bi-interfacial bond for enhancing the mechanical and fracture properties, which are important properties for their industrial potential. A large lumen enhances the ability of SHNTs to be used as nanocontainers for liquids and gas barriers in industrial, medical and thermal resistance applications. The curvature and bending without breaking properties of SHNTs help enhance their mechanical properties and fracture resistance. A long SHNT length creates the ability to bend and curve. Transparency due to the large lumen and thin SHNT walls helps in nanocontainer applications and may be suitable for medical applications where the filling materials in the SHNTs can be seen and checked. Furthermore, the SHNT transparency is suitable for the cosmetics industry. For industrial and medical applications, for example, adding a small ratio of SHNTs (3–5%) to reinforce the polymer matrix will enhance mechanical and fracture properties in addition to thermal properties by controlling and preventing degradation of the CPNC. SHNTs can dramatically enhance the thermal properties of a CPNC, where it can help save energy and water consumption in arid areas, such as Saudi Arabia.

## Conclusion

Advantages of newly explored nanotube Saudi clay show that it is transparent, while other types are opaque; SHNTs do not agglomerated, while others clay materials do. Kaolin clay layers agglomerate and are easy to exfoliate, while other types of clay require complicated processing. Additionally, SHNTs have a high surface area and can be used as nanocontainers and nanoreactors because they do not swell or shrink. The cross-section of the newly explored nanoclay is not completely circular, but may be nearly circular, rectangular, octagonal, hexagonal, or pentagonal. These shapes make SHNTs bind tightly to the polymer in a CPNC, especially with the high surface area of SHNTs. SHNTs are shorter than conventional HNTs, which means that this newly explored nanoclay is better than conventional halloysite during processing. Furthermore, SHNTs are almost always straight without a high curvature. Therefore, based on these properties, SHNTs exhibit no fractions or breaks during processing, while HNTs can easily be broken. Finally, SHNTs have a very special transparency phenomenon and a high surface area.

## Methods

New Saudi halloysite-like nanotubes SHNTs have been discovered through detailed characterization and testing. Essential characterizations were carried out to investigate the microstructure, geometry, dimensions, shape, curvature, cross-section and transparency using scanning electron microscopy (SEM), as shown in Fig. 1, and transmission electron microscopy (TEM), as shown in Fig. 2. The chemical composition was also investigated using both SEM and TEM, as indicated in Table 1. Using the available laser particle size analyser in our laboratory, which is only a wet analysis device unit with a minimum measurement accuracy of 100–20,000 nm, the results are compared to MMT and Saudi Kaolinite clay, as indicated in shown in Fig. 3. Since the surface area is an important parameter for nanoparticles, controlling the electrical charges of particles, bonding interfaces, water absorption and swelling-shrinking characteristics, the Langmuir standard technique was used to investigate the surface area of SHNTs and compare it to HNTs in addition to agglomerated and exfoliated MMT. The surface electrical charge of SHNTs is investigated using the zeta potential technique, and the results are comparison to other Saudi kaolin clays, as shown in Fig. 4. Since SHNTs are nanopowders, it is difficult to separately characterize the mechanical, fracture and thermal properties for the powder shape directly. Therefore, some other tests were carried out to check these properties indirectly by changing the phase of the SHNTs powder to the solid phase by mixing it with other materials and checking the changes in the properties of the final product through standard tests. Therefore, an advanced CPNC material is produced by using 95% HDPE with an additive of 5% SHNTs. Then, it is followed by investigating the effect of SHNTs on changing each of the mechanical and fracture properties, as shown in Table 2 and Fig. 5. To investigate the thermal resistance of SHNTs, a packaging system made of sheets filled with dry SHNT powder was used for insulating full water tanks exposed directly to atmospheric temperature changes during the day and checked at specific times to observe the effect of SHNTs in comparison to non-insulated water tanks under the same climate changes; the results are shown in Table 2 and Fig. 6. Currently, Saudi Arabia is suffering from high temperature changes with arid areas; these areas require water cooling on summer days and heating in winter. Thus, the use of SHNT insulation without electrical energy will save water and energy. Detailed analysis is mentioned in the results and discussion sections.

*Scanning electron microscopy (SEM)*, the study methods included using the SEM equipment of where the detailed characterization was carried out fundamentally to study the newly discovered SHNTs.

*Transmission electron microscopy (TEM)*, as another important equipment had the most important mail role in discovering and characterizing SHNTS is the TEM which could investigate the most important aspects of the microstructural morphology.

*The energy dispersive analysis of X-rays (EDX)*, of both SEM and TEM equipment could explore successfully the chemical composition of SHNTs matching and proving each other as shown in Table 1.

*Particle size analysis of SHNTs*, it is studied by laser particle size analyser equipment to investigate the dimensions of SHNTs which may be out of the range of ability of SEM and TEM measurements. The measurements included comparison to other available types of nanoclay particles like MMT and kaolinite. Figure 3 shows the results and comparison indicating the relation between particle size (X μm) and particle size percentage [Q3(X) %] for each of MMT (Fig. 3a), Kaolinite (Fig. 3b) and SHNTS (Fig. 3c). the comparison shows that the mean particles size of the particles at 10% of test samples for each of MMT, kaolinite and SHNTs are 3200, 1400 and 900 nm respectively while at 50% of the tested samples amounts are 11,000, 4800 and 3700 nm respectively. In the same time the dimensions of mean size at 90% are 25,000, 11,000 and 9700 nm respectively for MMT, kaolinite and SHNTS.

*Surface area of SHNTs*, is tested using the standard Langmuir method in comparison to MMT and conventional halloysite HNT.

*Zeta potential (ZP) for SHNTs*, particle agglomeration or separation depends on the efficiency of bonding or debonding to other particles due to the amount of electrical charge on the surface of the particles. Additionally, the ability of the particles to absorb or lose water depends on the presence of free electrical charge on particle surfaces. The electrical charge is also responsible for the swelling and shrinking of nanoparticles. The stability of nanoparticles can be controlled by the amount of electrical charge on the particle surface where stable particles will not agglomerate, fluctuate or absorb a large amount of water; thus, these particles will not swell. Therefore, the electrical charge of SHNTs were measured by the zeta potential (ZP) technique and compared to other clay particles that swell, such as kaolinite clay. The ZP represents a good measurement scale of the cation exchange capacity (CEC) of the SHNTs, thereby investigating the energy capacity for bonds between particles.

*Mechanical and fracture properties of SHNT-based CPNCs*, because it is difficult to measure the mechanical and fracture properties of an SHNT powder directly, it is checked by measuring its effects on changing and enhancing the mechanical and fracture properties of a additive in comparison to the same properties of samples made of a pure polymer matrix. The test is carried out for both pure HDPE and the CPNC made of HDPE and SHNT based on the ASTM standard D638^14^ for tensile test and the ASTM standard D5045^15^ for fracture test. The results of the tests are shown in Table 2 and Fig. 5, including the mechanical test for tensile strength (σ test) and other mechanical properties, such as Young’s modulus of elasticity (E) and Poisson’s ratio (ν), for both pure HDPE and the CPNC made of HDPE and SHNT based on the ASTM standard D638^14^.

*Finally, thermal resistance of SHNTs*, as an application for insulation, we already carried out experiments using a dry SHNT powder for insulating water tanks to inhibit changes in the water temperature without the use of electrical heating or cooling, as shown in Fig. 6. To check the thermal resistance capacity of SHNTs, special thin sheets were prepared and filled with a dry SHNT powder covering the small HDPE water tanks exposed to the normal temperatures of the arid Saudi climate during the day and night. The water temperature was measured in addition to the surrounding temperature changes and compared to the water temperature of the reference water tank without insulation.

### Sample preparation

Characterization and testing include scanning electron microscopy (SEM), transmission electron microscopy (TEM), energy dispersive analysis of X-rays (EDX) zeta potential (ZP) and particle size analysis. Additionally, the mechanical, fracture and thermal properties of SHNTs were analysed in specific applications. SHNTs samples for characterization were prepared in the form of dry powder for SEM, and the microstructure, dimensions, particle distribution and arrangement, particle shape and geometry, curvature, cross-section and elemental composition were characterized through the use of SEM–EDX. A small amount of SHNTs were fixed on small discs for the SEM device by using double-sided carbon tape. Then, these samples were coated by gold or carbon using a plasma sputtering device before installing the test discs inside the test chamber of the SEM device for characterization. To characterize the chemical composition by SEM, the samples were installed in the SEM device without any coating. TEM–EDX characterization included investigations on the microstructure, particle transparency, cross-section, lumen, shape, dimensions, geometry, curvature and elemental composition. Samples for TEM characterization were difficult to prepare and fix on the characterization grid without using fixing tape because the grid was very small. Thus, it was very difficult to prepare dry SHNT samples. Therefore, a small amount of SHNTs was mixed in distilled water to produce an SHNT suspension. Then, a small drop of the SHNT suspension was dropped on the small TEM grid and left to dry. Next, the grid was installed inside the test chamber of the TEM device for characterization. Samples for the zeta potential (ZP) test were prepared with small amounts of dry SHNTs mixed with distilled water in the testing cell, and then the cell was installed in the ZP device chamber. The particle size was analysed by a wet unit laser particle size analyser using small samples. The available particle size device was only accurate at the microscale; thus, the smallest size that could be measured was 100 nm. In regard to the thermal test, thin sheets of hollow columns with diameters of 1–2 cm were prepared and filled with dry SHNT powder for use as a packaging layer in water tanks to check their ability to inhibit water temperature changes. This test was conducted in a thermally controlled room in the laboratory under constant temperature and in an open-air field at various temperatures during the day and night. The thermal test results are shown in Fig. 6.

### Applications of SHNTs

SHNTs will be important in industrial and medical applications, similar to HNTs^3^, in addition to environmental applications. SHNTs will be used for the development of advanced nanocomposites, enhancing the mechanical properties along with the fracture resistance and thermal resistance of materials, such as polymers for different industrial applications, namely, nanocontainers, gas barrier materials, packaging, automobiles, electronics, fire retarding, thermal resistance and insulation materials.

## Data Availability

All data and results are available upon request.
